# Influence of Repetitive Transcranial Magnetic Stimulation on Human Neurochemistry and Functional Connectivity: A Pilot MRI/MRS Study at 7 T

**DOI:** 10.3389/fnins.2019.01260

**Published:** 2019-11-27

**Authors:** Heidi Gröhn, Bernadette T. Gillick, Ivan Tkáč, Petr Bednařík, Daniele Mascali, Dinesh K. Deelchand, Shalom Michaeli, Gregg D. Meekins, Michael J. Leffler-McCabe, Colum D. MacKinnon, Lynn E. Eberly, Silvia Mangia

**Affiliations:** ^1^Department of Radiology, Center for Magnetic Resonance Research, University of Minnesota, Minneapolis, MN, United States; ^2^Diagnostic Imaging Center, Kuopio University Hospital, Kuopio, Finland; ^3^Department of Rehabilitation Medicine, University of Minnesota, Minneapolis, MN, United States; ^4^Department of Biomedical Imaging and Image-guided Therapy, High Field MR Centre, Medical University of Vienna, Vienna, Austria; ^5^Museo Storico della Fisica e Centro Studi e Ricerche “Enrico Fermi”, Rome, Italy; ^6^Department of Neurology, University of Minnesota, Minneapolis, MN, United States; ^7^Division of Biostatistics, School of Public Health, University of Minnesota, Minneapolis, MN, United States

**Keywords:** GABA, repetitive transcranial magnetic stimulation, magnetic resonance spectroscopy, resting-state functional MRI, motor cortex, inhibition, non-invasive brain stimulation, functional connectivity

## Abstract

Repetitive transcranial magnetic stimulation (rTMS) is a non-invasive brain stimulation method commonly used in the disciplines of neuroscience, neurology, and neuropsychiatry to examine or modulate brain function. Low frequency rTMS (e.g., 1 Hz) is associated with a net suppression of cortical excitability, whereas higher frequencies (e.g., 5 Hz) purportedly increase excitability. Magnetic resonance spectroscopy (MRS) and resting-state functional MRI (rsfMRI) allow investigation of neurochemistry and functional connectivity, respectively, and can assess the influence of rTMS in these domains. This pilot study investigated the effects of rTMS on the primary motor cortex using pre and post MRS and rsfMRI assessments at 7 T. Seven right-handed males (age 27 ± 7 y.o.) underwent single-voxel MRS and rsfMRI before and about 30-min after rTMS was administered outside the scanner for 20-min over the primary motor cortex of the left (dominant) hemisphere. All participants received 1-Hz rTMS; one participant additionally received 5-Hz rTMS in a separate session. Concentrations of 17 neurochemicals were quantified in left and right motor cortices. Connectivity metrics included fractional amplitude of low-frequency fluctuations (fALFF) and regional homogeneity (ReHo) of both motor cortices, strength of related brain networks, and inter-hemispheric connectivity. The group-analysis revealed few trends (i.e., uncorrected for multiple comparisons), including a mean increase in the concentration of the inhibitory neurotransmitter γ-aminobutyric acid (GABA) after the inhibitory rTMS protocol as compared to baseline in the stimulated (left) motor cortex (+8%, *p* = 0.043), along with a slight increase of total creatine (+2%, *p* = 0.018), and decrease of aspartate (−18%, *p* = 0.016). Additionally, GABA tended to decrease in the contralateral hemisphere (−6%, *p* = 0.033). No other changes of metabolite concentrations were found. Whereas functional connectivity outcomes did not exhibit trends of significant changes induced by rTMS, the percent changes of few connectivity metrics in both hemispheres were negatively correlated with GABA changes in the contralateral hemisphere. While studies in larger cohorts are needed to confirm these preliminary findings, our results indicate the safety and feasibility of detecting changes in key metabolites associated with neurotransmission after a single 1-Hz rTMS session, establishing the construct for future exploration of the neurochemical, and connectivity mechanisms of cortical responses to neuromodulation.

## Introduction

Repetitive transcranial magnetic stimulation (rTMS) is a non-invasive brain stimulation method commonly used in the disciplines of neuroscience, neurology, and neuropsychiatry to examine or modulate brain function. The effects of rTMS are transient, and critically dependent upon the location, frequency and intensity of stimulation. Low frequency rTMS (e.g., 1 Hz) is associated with a net suppression of the excitability of cortical structures beneath the site of stimulation while higher frequency stimulation (e.g., 5 Hz) has been shown to increase net cortical excitability. rTMS has been shown to have potential efficacy for treating both psychiatric and non-psychiatric disorders (Machado et al., 2008), such as posttraumatic stress disorder, obsessive compulsive disorder, auditory hallucinations in schizophrenia, pain syndromes, and for improving motor function in neurodegenerative diseases or following stroke. rTMS is currently FDA approved for the symptomatic relief of treatment-resistant depression. Despite the increasing development and use of rTMS in research and clinical applications, its mechanisms of action are still relatively poorly understood. Studies in animals and humans have provided evidence that rTMS protocols can influence the excitability and function of neurons (neuromodulation), both near to, and distant from, the site of stimulation (Dayan et al., 2013; Liew et al., 2014; Huang et al., 2017). While it is still unclear how local and distant changes in function induced by specific rTMS protocols are mediated, changes in the levels of excitatory and inhibitory neurotransmitters, glutamate and γ-aminobutyric acid (GABA), respectively, most likely play a critical role in neuromodulation effects.

Recent advancements in functional MRI (fMRI) (Van Essen et al., 2012; Smith et al., 2013; Glasser et al., 2016) and functional magnetic resonance spectroscopy (fMRS) (recently reviewed in Jelen et al., 2018; Stanley and Raz, 2018) have allowed monitoring functional connectivity and neurochemistry in the human brain with exceptional sensitivity and robustness. We showed that an extensive number of brain metabolites can be measured non-invasively in the human brain at ultra-high magnetic field of 7 T (Tkáč et al., 2001). We subsequently demonstrated that changes in neural activity are associated with relatively small changes in several metabolite concentrations which could be reliably detected at 7 T (Mangia et al., 2007a, b; Bednařík et al., 2015, 2018), as confirmed by other labs (Lin et al., 2012; Schaller et al., 2013, 2014; Boillat et al., 2019). The capability to measure these functional changes provides unique insights into how brain metabolism is associated with neural activity (Mangia et al., 2009). Importantly, extensive efforts are currently focused on optimizing methodologies of edited MRS at 7 T for further enhancing the reliability of detection of the inhibitory neurotransmitter GABA (Chen et al., 2017; Hendriks et al., 2018).

The majority of studies exploring the effect of rTMS on neurochemical concentrations have been performed after multiple sessions of rTMS on prefrontal cortex for applications mostly in depressive disorders, and also in schizophrenia and addiction (Zheng et al., 2010; Croarkin et al., 2016; Dubin et al., 2016; Qiao et al., 2016; Dlabac-de Lange et al., 2017; Hone-Blanchet et al., 2017; Bridges et al., 2018). High frequency (10–20 Hz) stimulation resulted in significant metabolic changes, however, findings showed large variability likely due to different stimulation protocols, patient populations and brain areas from which MR spectra were acquired. In healthy volunteers, an increase in the glutamate plus glutamine (Glx) level was observed both near the stimulation site and in remote brain regions after single and series of consecutive rTMS sessions (Michael et al., 2003). In another study, 5 weeks of 10-Hz rTMS induced increase in GABA in medial prefrontal cortex with no significant effect on Glx in depressive disorder patients (Dubin et al., 2016). However, 3 weeks of 10-Hz rTMS was found in another study induced an increase of Glx levels in the left dorsolateral prefrontal cortex of treatment responders (Yang et al., 2014). Notably, only two previous studies have investigated the acute effects of a single session of rTMS, applied to the motor cortex, on neurochemical concentrations. A local decrease of NAA in the stimulated motor cortex was found in healthy adult control volunteers as well as in adults with dystonia after 8 min of 5-Hz rTMS, while GABA slightly increased in the controls and slightly decreased in the adults with dystonia (Marjanska et al., 2013). Similarly, Stagg et al. (2009b) showed an increase in GABA levels of the stimulated site up to 20 min after a short 40-s period of continuous theta burst stimulation applied to healthy controls, without significant change in Glx.

Considering this variability, and the gaps in understanding of the mechanisms of action of rTMS, the primary aim of this pilot study was to assess the feasibility of detecting rTMS-induced effects on high-resolution functional connectivity metrics and neurochemicals measured at 7 T with state-of-art methodologies. In particular, we chose to acquire the MRS data with semi-LASER sequence for robust, simultaneous measurement of the full neurochemical profile, including excitatory (glutamate) and inhibitory (GABA) neurotransmitters. Our main focus was the investigation of a 1-Hz inhibitory rTMS intervention applied outside the scanner for 20 min over the primary motor cortex of the presumed dominant hemisphere. We hypothesized that rTMS would induce changes in the neurochemical profiles and functional outcomes, and that trends of such changes could be detected at 7 T. Specifically, we expected that a low-frequency rTMS protocol (1-Hz rTMS) that typically suppresses motor cortical excitability for 15 to 60 min (Chen et al., 1997; Hoogendam et al., 2010) would be associated with increased GABA concentration in the ipsilateral hemisphere. Since 1-Hz rTMS also induces a lasting suppression of GABAergic interhemispheric inhibition (Pal et al., 2005), we also hypothesized that low frequency rTMS would be associated with decreased GABA and increased glutamate in the contralateral motor cortex.

An additional objective of this study was to assess the safety of the study design which combines two 7 T MRI sessions acquired immediately before and after, respectively, a prolonged rTMS intervention. In fact, similar pre- and post- assessment designs have been used safely at 3 T with rTMS of shorter duration (Stagg et al., 2009b; Marjanska et al., 2013). Ultra-high field MRI has been also used in combination with TMS assessment of cortical excitability (Dyke et al., 2017). However, in that study no post-assessment MRI was performed, and only single and paired-pulse TMS was applied which, unlike rTMS, do not evoke lasting changes in corticomotor excitability. For this reason, we could not exclude the possibility of potential interaction effects between the two 7T MRI sessions and the rTMS intervention.

## Materials and Methods

### Subject Characteristics and Consent

Seven adult (21 – 40 y.o.), healthy, right-handed males were recruited for the study. They all received 1-Hz rTMS, and one additionally received 5-Hz rTMS in a separate session, 3 weeks after the 1-Hz protocol. Exclusion criteria included females (to avoid unknown risks on the unborn fetus of multiple 7 T MRI sessions and a prolonged rTMS intervention combined in one study visit), contraindications to MRI, diagnosis of a psychiatric disorder, substance abuse, epilepsy, neurological and/or cardiovascular disease, head trauma that may have caused traumatic brain injury, brain tumor or stroke, sleep apnea, history of anxiety, syncope, panic attacks and/or claustrophobia, being currently on any medication. The study protocol was approved by the Institutional Review Board: Human Subjects Committee of the University of Minnesota in accordance with the recommendations of The Code of Federal Regulations and the Declaration of Helsinki. A written informed consent was provided by each subject right before the beginning of the study. The study was registered on ClinicalTrials.gov (NCT02677740).

### Experimental Design

The study consisted of one study visit during which resting state fMRI and MRS data were collected at 7 T before and after the rTMS intervention. The study started with the pre-rTMS MRI session, during which metabolite concentrations and functional connectivity outcomes were first measured at baseline. ^1^H MRS data were acquired from the volumes of interest (VOIs) located in the left and right motor cortices based on anatomical landmarks of high-resolution MRI. After the pre-rTMS imaging session finished, the participant was transferred from the magnet to a room with the equipment for rTMS. The subject rested for about half an hour to 1 hour, which was deemed sufficient for minimizing possible effects of the exposure to the high magnetic field on cortical excitability (Schlamann et al., 2010). First, motor evoked potentials (MEP) were measured by delivering TMS, then the 20-min rTMS intervention was administered, and then the MEP were measured again. At the end of the rTMS session, the subject was directly returned to the MR scanner room for the post-rTMS MRI/MRS session, where great care was applied to ensure that the MRS VOIs were selected as close as possible to those of the pre-rTMS session. For both pre- and post-rTMS sessions, data were acquired in the following order: anatomical MRI, MRS from left then from right motor cortex and finally rsfMRI. Given the transitory nature of neuromodulatory effects, we did not randomize the MRI acquisition order to minimize the uncertainty of the post-assessment timings. Participants were instructed to remain awake during both rsfMRI and MRS acquisitions, and to keep their eyes closed during the rsfMRI acquisitions. Operator-participant verbal contact was maintained during the MRI sessions using the intercom system to verify compliance, wakefulness and comfort level.

### Safety Assessments and Feasibility Metrics

Recruitment status (i.e., number of drop-outs and incomplete datasets), safety issues, symptoms questionnaires, and protocol timings (i.e., post-assessment delays from the rTMS intervention) were recorded. Blood pressure and heart rates were also measured within 5 min before and after the rTMS intervention. Blood oxygenation and pulse rate were additionally measured with an MRI-compatible oximeter during the MRI sessions to monitor vital signs. Each participant was evaluated for any adverse event throughout the study. A medical director was present throughout the rTMS session and the second imaging session. The medical director reviewed all participant outcomes including responses to report of symptoms questionnaires after the MRI and rTMS sessions, along with the vital signs (blood pressure and heart rate before rTMS, blood oxygenation, and heart rate during the MRI scan), and approved continuation of the study after each session. A designated medical monitor was assigned to review any adverse event that occurred.

### TMS, rTMS, and Neurophysiological Outcomes

TMS was delivered using a 70-mm figure-eight TMS coil connected to a Magstim 200 machine. The coil was positioned over the hand region of the left motor cortex (approximately 5 cm lateral to each individual vertex of the head), contralateral to the dominant hand in all studied participants. The coil was oriented to a position tangential to the scalp and with the handle pointing posterolaterally at an approximately 45-degree angle to the sagittal line. TMS-induced MEPs were recorded by placing surface EMG electrodes over the first dorsal interosseous muscle of the participant’s dominant hand, contralateral to the side of TMS. Stimulation was set to an initial intensity to evoke a discernable MEP amplitude. The position of the coil was moved systematically to find the location that evoked the largest and most reliable MEP (“hotspot”).

The resting motor threshold (RMT) was the primary neurophysiologic outcome, and was defined as the minimum TMS intensity required to produce a 50 μV MEP while the participant was at rest. RMT was determined by progressively lowering the intensity until MEPs ≥ 50 μV peak-to-peak were evoked in at least 5 of 10 successive stimuli.

Immediately after completion of the RMT assessment, the rTMS intervention began. Participants received either 1-Hz (7 subjects) or 5-Hz (1 subject) rTMS, applied over the motor cortex hotspot contralateral to the dominant arm for 20–22 minutes at an intensity of 90% RMT (1-Hz rTMS: train of 10 pulses, 1 s wait time between trains, 120 trains, total pulses = 1200; 5-Hz rTMS: train of 25 pulses, 45 s wait time between trains, 24 trains, total pulses = 600). This number of stimuli were well within the published safety guidelines for use of rTMS (Wassermann, 1998). Corticospinal excitability was finally re-tested within no more than 10 min after the rTMS session using the same protocol as the pre-test.

### MRI/MRS Acquisitions

The imaging sessions were performed using a 7-T/90-cm magnet (Agilent/Magnex Scientific, United Kingdom) equipped with a powerful gradient/shim coil (SC72, Siemens, Germany; maximum gradient strength of 70 mT/m; maximum second-order shim strength of 4.5 mT/m^2^ except for Z^2^ which was about 9 mT/m^2^) and interfaced to a Siemens Syngo console. A single channel transmit/32-channel receive head (NOVA Medical, Wilmington, MA, United States) was used in combination with dielectric pads (Teeuwisse et al., 2012; Schaller et al., 2014). High permittivity water/titanate-based (30–50% v/v) dielectric pads (5–8 mm thick) were carefully placed on the participant’s head above the central sulcus, so that each pad covered the primary motor cortex on one hemisphere. This approach enabled to generate B_1_ over 25 μT within the VOI in the motor cortex. Head motion was minimized using a molded foam head cushion and padding placed snugly around the participant’s head.

**FIGURE 1:**
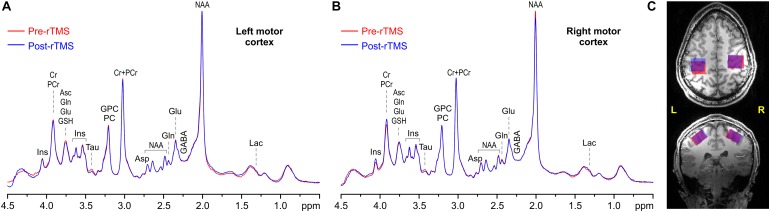
Representative example spectra acquired pre (red) and post 1-Hz rTMS (blue) from the left **(A)** and right **(B)** VOIs, along with VOI placements **(C)** in the motor cortices in axial (top) and coronal (bottom) view (overlap is in purple). MRS acquisition parameters: 7 T, semi-LASER, 64 scans, TR = 9 s, and TE = 28 ms.

High-resolution 3D-MPRAGE images (TR = 2.5 s, TE = 2.42 ms, TI = 1.5 s, flip angle = 5^*o*^, isotropic resolution = 1 × 1 × 1 mm^3^, acquisition time of 5 min) were first obtained to visualize the anatomical structure of the motor cortices. Proton density images (TR = 1.42 s, TE = 2.42 ms, flip angle = 5^*o*^, isotropic resolution = 1 × 1 × 1 mm^3^, acquisition time of approximately 1.5 min) were also acquired as reference to allow normalization of the T_1_-weighted signal intensities of MPRAGE images across the field of view (Van de Moortele et al., 2009). Then automatic B_0_ field mapping and adjustment of 1st- and 2nd-order shims were achieved by FASTMAP with EPI readout (Gruetter and Tkáč, 2000). Spectroscopy VOIs (24 × 22 × 17 mm^3^) were selected in left and right motor cortices based on anatomical landmarks (Figure 1). ^1^H-MRS data were acquired using the semi-LASER localization sequence optimized for 7 T (Oz and Tkáč, 2011; Bednařík et al., 2015) with the gradient-modulated FOCI pulses for reducing the demands on maximum B_1_ (TE = 28 ms, TR = 9 s, 64 scans, spectral width 6 kHz). The localization sequence included VAPOR water suppression interleaved with the outer-volume saturation (Tkáč and Gruetter, 2005). Unsuppressed water signal was also acquired from each VOI, which has been used for eddy current correction (Klose, 1990) and as an internal reference for metabolite quantification.

At the end of the MRS acquisitions, the dielectric pads were removed, T_1_-weighted anatomical scans acquired again, and resting state fMRI performed with a 2D single-shot gradient echo EPI sequence to collect blood oxygenation level dependent (BOLD) data. Acquisition parameters were as follows: FOV = 208 × 208 mm^2^, matrix = 104 × 104, 72 slices, slice thickness = 2 mm (resolution 2.0 × 2.0 × 2.0 mm^3^), TR/TE = 1200/20 ms, flip angle = 45°, BW = 2003 Hz/pixel, iPat = 2, multiband factor = 3. A total of 280 volumes were acquired, corresponding to an acquisition time of approximately 6 min. Spin Echo based field maps were also collected for correction of image distortions (Smith et al., 2013; Sotiropoulos et al., 2013).

### MRI/MRS Data Processing

Single-scan MRS data were corrected for small frequency and phase variations, summed and finally corrected for the residual eddy current effects using unsuppressed water signal (Klose, 1990). Brain metabolites were quantified by LCModel (Provencher, 1993, 2001; Pfeuffer et al., 1999; Tkáč et al., 2009) using a basis set of nineteen brain metabolites simulated with a spin density matrix approach (Henry et al., 2006), which included: alanine (Ala), ascorbate (Asc), aspartate (Asp), creatine (Cr), γ-aminobutyric acid (GABA), glucose (Glc), glutamate (Glu), glutamine (Gln), glutathione (GSH), glycerophosphocholine (GPC), *myo*-inositol (Ins), *scyllo*-inositol (sIns), lactate (Lac), N-acetylaspartate (NAA), N-acetylaspartylglutamate (NAAG), phosphocholine (PC), phosphocreatine (PCr), phosphoethanolamine (PE), and taurine (Tau). In addition, a spectrum of fast-relaxing macromolecules (MM) was also included in the basis set. Intensities of singlet resonances (CH_3_ groups) in the basis set spectra of NAA, NAAG, GPC, PC, Cr, and PCr were corrected for differences in T_2_ signal attenuation at TE = 28 ms between CH_3_ groups (*T*_2_ = 95 – 152 ms) relative to CH and CH_2_ groups within the same molecule (*T*_2_ = 84 – 95 ms) (Marjanska et al., 2012). High-resolution MRI data were used for 3D whole brain segmentation. The probabilistic maps of the gray matter (GM), white matter (WM), and cerebrospinal fluid (CSF) were calculated with SPM12^1^ (Ashburner and Friston, 2005) from the T_1_-weighted MPRAGE images that were normalized using the proton density reference (Van de Moortele et al., 2009). An in-house routine written in MATLAB R2009 was used to determine the volume fractions of GM, WM, and CSF in each VOI by adopting an iterative method of threshold selection (Ridler and Calvard, 1978). The CSF fraction was used to assess the brain tissue volume for each VOI selected for MRS. The tissue water content was calculated using GM and WM volume fractions in the VOIs and GM and WM water contents of 84% and 70%, respectively (Randall, 1938). Metabolite concentrations were additionally corrected for T_2_-relaxation. Relaxation times of water (*T*_2_ = 47 ms) and metabolites (*T*_2_ = 90 ms, average value for CH and CH_2_ groups) in occipital lobe (Marjanska et al., 2012) were multiplied by the factor 1.2 to correct for the CPMG effect of the semi-LASER RF pulse train. Only metabolite concentrations quantified with Cramèr-Rao lower bounds (CRLB) below 50% were included in further analysis. Finally, the overlap between pre- and post-rTMS VOI selection was evaluated, and quantification correlations among each metabolite as calculated in LCModel were extracted for all 4 spectra of one representative subject.

### rsfMRI Data Processing

Resting-state data were preprocessed with the HCP “minimal-preprocessing pipeline” (Glasser et al., 2013), and denoised with the FMRIB’s independent component analysis (ICA)-based X-noiseifier (FIX, Salimi-Khorshidi et al., 2014), which removes slow drifts (via a gentle high-pass filter with a 2000 s cutoff), motion-derived parameters (24 motion time series constructed from the 6-realignment parameters) and structured noise. We additionally regressed out the global signal (Burgess et al., 2016) and applied a low-pass filter (0.09 Hz cutoff) to remove non-structured and high frequency noise, respectively. Finally, the volume data were smoothed with an isotropic Gaussian kernel at 4 mm FWHM.

**FIGURE 2:**
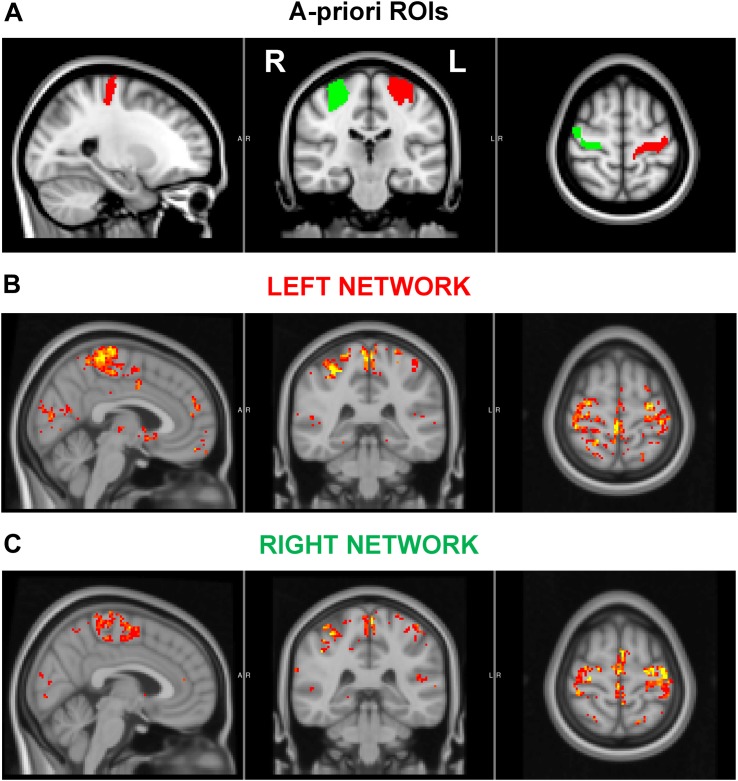
**(A)** *A priori* regions of interest (ROIs). **(B–C)** Associated left **(B)** and right **(C)** motor cortex networks used in functional connectivity analyses. The ROIs encompassed the upper limb region of the precentral gyrus, as defined in the Brainnetome atlas. The displayed networks were calculated at a group level as described in the text.

Resting-state activity (Figure 2) was quantified by examining various parameters, including the fractional amplitude of low-frequency fluctuations (fALFF, Zou et al., 2008), the regional homogeneity (ReHo, Zang et al., 2004), the global strength of the networks arising from the motor cortices, and the inter-hemispheric connectivity between the motor cortices. In particular, fALFF, which is thought to reflect the intensity of spontaneous brain activity, was calculated via AFNI function 3dRSFC (Cox, 1996) as the ratio of the power spectrum within the frequency range of interest (0.008 < *f* < 0.09 Hz) to that of the entire spectrum. ReHo, a measure of local synchronization, was calculated as the Kendall’s coefficient of concordance among a given voxel time series and the time series of its 18 nearest neighbors (3dReHo, AFNI). fALFF and ReHo were computed for each voxel in the brain and were then averaged within the left and right motor cortices, separately. These two regions of interest (ROIs) were identified in the upper limb region of the precentral gyrus, as defined in the Brainnetome atlas (Fan et al., 2016). The strength of the network, arising from either the ipsilateral or contralateral motor cortices, was obtained by first computing the seed-based connectivity map as the Pearson’s correlation between the average time series within the seed (either the left or right ROI) and the time series at each other voxel within the brain. Then, for each subject and seed, the network strength was defined as the average of the z-Fisher-transformed connectivity map within a group-level network, obtained via a one-sample, one-tail, *t*-test on the connectivity maps of the pre and post-intervention data (*p* < 0.001, minimum cluster size 20). The inter-hemispheric connectivity was calculated as the z-fisher-transformed Pearson’s correlation between the average time-courses of right and left ROIs. Finally, the framewise displacement as defined in Power et al. (2012) was computed to assess head movements during functional acquisitions.

### Statistical Analyses

VOI overlaps, VOI tissue compositions, signal-to-noise ratios (SNRs), and spectra linewidths were first subjected to comparisons with 2-sided paired *t*-test to ensure the absence of systematic biases in the datasets induced by VOI placements left vs. right, and pre- vs. post-rTMS where applicable (i.e., except VOI overlaps). Pre-rTMS metabolite concentrations were also compared between left and right motor cortices with 2-sided paired *t*-tests for further characterizing neurochemical profiles at baseline. Pre- vs. post-rTMS comparisons of metabolite concentrations and functional connectivity metrics were carried out with 2-sided paired *t*-test separately for left and right motor cortices where applicable (i.e., except inter-hemispheric connectivity). Given the nature of the study as a pilot investigation, multiple testing corrections to type I error were not applied. Finally, exploratory Pearson correlation analyses were performed between percent changes of connectivity metrics and MRS outcomes (namely glutamate and GABA concentrations). Since only one participant received the 5-Hz rTMS, analyses of rTMS-induced changes and their correlations were performed only on the 1-Hz rTMS dataset (*N* = 7). Results are presented as mean ± SD, and Pearson correlations are reported with 95% CI.

## Results

### Safety, Feasibility, and Neurophysiological Outcomes

All recruited participants (ages 27 ± 7 y.o.) completed the study and provided a complete and usable dataset. Symptoms reported after each 7T MRI session were expected, occasional, and did not increase after the rTMS intervention. In particular, after the first MRI session, one participant reported metallic taste, one sleepiness and warmth, and one sleepiness and metallic taste. After the second MRI session, two participants reported sleepiness. The rTMS intervention did not increase symptoms reported prior to its administration. Indeed, one participant reported sleepiness, anxiety/worry/nervousness, headache, and tooth pain before and after the rTMS intervention. Two other participants reported either sleepiness, or sleepiness and anxiety/worry/nervousness, before but not after the rTMS intervention. Finally, one participant reported shoulder pain the day after the study. Vital signs were within physiological ranges during the entire study duration for all subjects.

There was no significant difference in the RMT before and after 1-Hz rTMS (mean change = 4.4 ± 13.4%; *p* = 0.416). Four participants showed an increase in RMT after receiving 1-Hz rTMS, which is consistent with a decrease in cortical excitability, but 3 participants showed an average decrease in motor threshold. In the participant that received 5-Hz rTMS, a 36% increase in motor threshold was observed. The delays of MRI/MRS post-assessments from the end of the rTMS intervention were (32 ± 3) min for MRS in left VOI, (59 ± 6) min for MRS in right VOI, and (84 ± 8) min for rsfMRI.

### MRS Outcomes

Overlap between pre-rTMS and post-rTMS VOIs tended to be slightly higher in right (91 ± 4)% vs. left (85 ± 7)% motor cortex (*p* = 0.035). On the other hand, VOI compositions included on average (7 ± 3)% CSF, (48 ± 6)% GM, and (44 ± 7)% WM, with no significant difference between left and right VOIs, or pre- and post-rTMS. The combination of high magnetic field, sensitive RF coil, use of dielectric pads, highly efficient B_0_ shimming, and full signal intensity semi-LASER localization sequence allowed obtaining artifact free spectra (Figure 1) with high signal-to-noise ratio (210 ± 27) and spectral resolution (FWHM = 10.2 ± 0.9 Hz, assessed from total Cr signal at 3 ppm) from all datasets, again with no significant differences between left and right VOIs, or pre- and post-rTMS.

**FIGURE 3:**
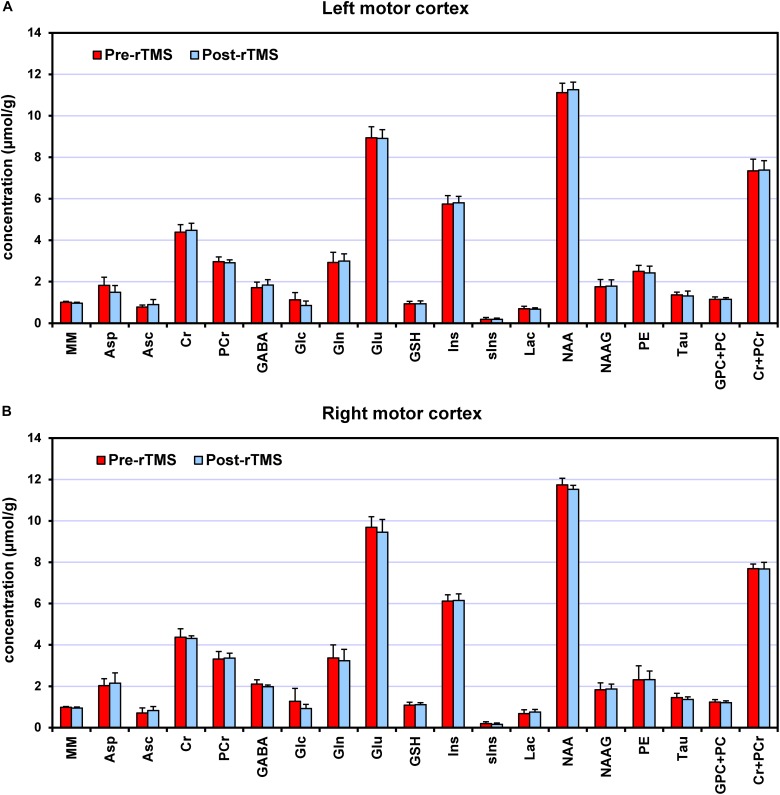
Neurochemical profiles pre-rTMS (red) and post-rTMS (blue), from left **(A)** and right **(B)** motor cortices. Bar diagrams show mean ± SD across the subjects who underwent the 1-Hz rTMS (*N* = 7).

Seventeen metabolites and the MM content were quantified (Figure 3) with average CRLB below 30%, among which Cr, PCr, GSH, Gln, Glu, *myo*-Ins, NAA, NAAG, PE, Tau and total choline (GPC + PC) and total creatine (Cr + PCr) had CRLBs below 10%. Macromolecules had 2% average CRLB. Only 4 out of 32 values of Glc and 5 out of 32 values of *s*Ins were excluded from the final analysis because of their CRLBs above 50%. The average CRLB of GABA and glutamate were 13% and 2%, respectively. In absolute terms, the values of CRLBs in concentration units were <0.2 μmol/g for most metabolites. In left motor cortex, the primary outcomes of interest, namely GABA and glutamate, had average concentrations of (1.70 ± 0.25) μmol/g (15% c.v.) and (8.89 ± 0.51) μmol/g (6% c.v.), respectively. Among the quantified metabolites, there was strong negative correlation between PCr and Cr, but both metabolites were consistently quantified in all subjects with very low CRLB (∼4%). Therefore, individual values could still be considered reliable. No negative correlations of GABA with any metabolite were observed. Correlation between GABA and MM was also negligible, ranging from −0.15 to −0.08.

At baseline (i.e., pre-rTMS), several metabolite concentrations had higher concentration in right vs. left motor cortex, including GABA (24%, *p* = 0.002), glutamate (8%, *p* = 0.003), glutamine (16%, *p* = 0.001), Cho (6%, *p* = 0.019), Ins (7%, *p* = 0.003), GSH (18%, *p* = 0.041), and NAA (6%, *p* = 0.011).

**FIGURE 4:**
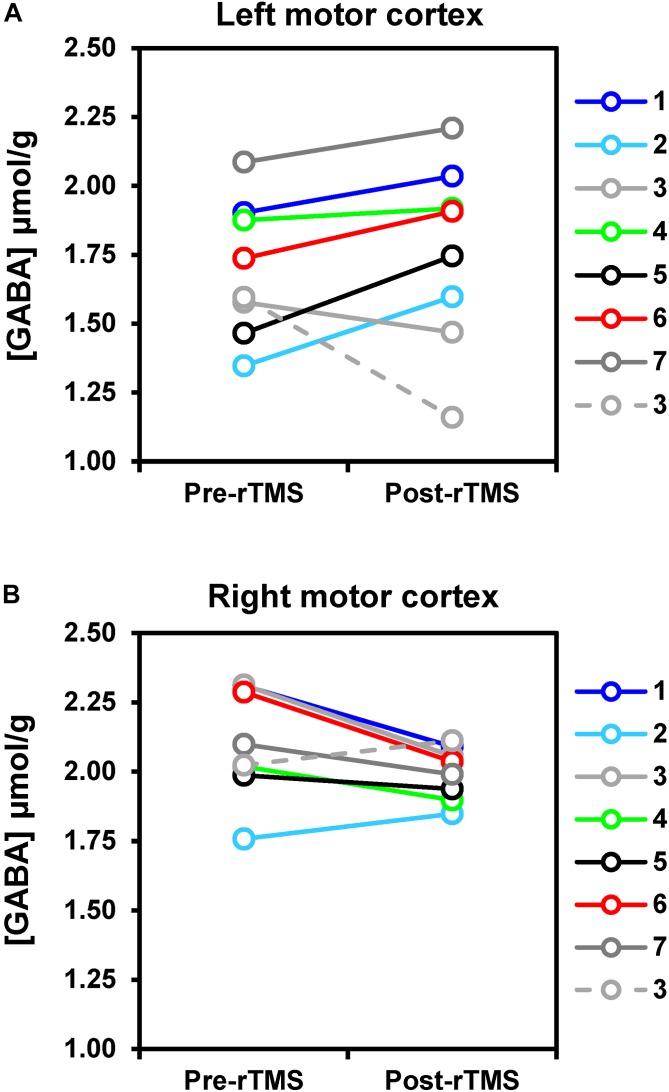
rTMS-induced changes of GABA concentrations in left **(A)** and right **(B)** motor cortices. Solid lines represent data obtained with 1-Hz rTMS intervention (*N* = 7), while the dashed line refers to the one subject (namely subj. 3) who received 5-Hz rTMS in a separate study visit. Changes in GABA levels were small but detectable with statistical significance at uncorrected *p*-value. In particular, GABA increased by 8% on average after rTMS as compared to pre-rTMS in the motor cortex ipsilateral to the stimulation (*p* = 0.043), whereas it decreased by 6% on average in the right motor cortex (*p* = 0.033).

After the 1-Hz rTMS intervention (Figure 4), GABA concentration in the ipsilateral (left) motor cortex increased on average by 8% as compared to pre-rTMS (*p* = 0.043), while in the contralateral (right) motor cortex, decreased by 6% (*p* = 0.033). Opposite changes in GABA were seen in the only subject who underwent the 5-Hz rTMS. No significant changes in glutamate levels were detected in either left or right motor cortices.

No other metabolites exhibited significant concentration changes, except Cr (2%, *p* = 0.016) and Asp (−18%, *p* = 0.018) in the ipsilateral motor cortex. To check for robustness to parametric assumptions, paired comparisons were repeated with non-parametric Wilcoxon signed-rank tests; conclusions were no different and these results are not presented.

### Functional Connectivity Outcomes

**FIGURE 5:**
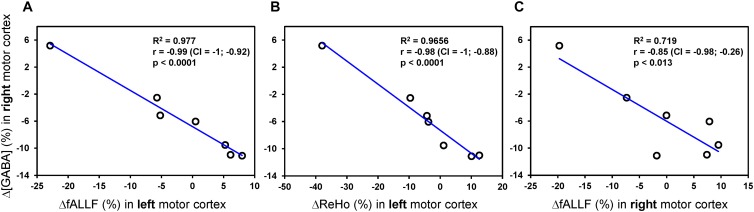
Pearson correlations analyses between rTMS-induced percent changes of MRS and connectivity metrics. Only Pearson correlations with *p* < 0.05 (uncorrected) are displayed. The plots show a strong linear relationship between percent changes of GABA concentration [Δ(GABA)] in contralateral (right) motor cortex and both ΔfALLF **(A)** and ΔReHo **(B)** in ipsilateral (left) motor cortex. A similar relationship, but with more variance, was seen with the ΔfALLF in contralateral (right) motor cortex **(C)**.

Head movements quantified by the framewise displacement were similar during pre-rTMS (0.17 ± 0.07 mm) and post-rTMS (0.16 ± 0.07 mm) rsfMRI acquisitions. None of the connectivity metrics showed significant rTMS-induced changes after the 1-Hz rTMS. However, as shown in Figure 5, percent changes in GABA concentration of the contralateral (right) motor cortex were negatively correlated with percent changes of fALFF in contralateral motor cortex (*r* = −0.85, *p* = 0.013, CI = −0.98; −0.26), as well as with percent changes of fALFF (*r* = −0.99, CI = −1; −0.92; *p* < 0.0001), and ReHo (*r* = −0.98, CI = −1; −0.88; *p* < 0.0001) in ipsilateral (left) motor cortex. No other significant correlations were observed.

## Discussion

In the present pilot investigation, we confirmed the feasible and safe integration of state-of-the-art MRS and rsfMRI at 7 T with rTMS interventions to measure changes in neurochemical profiles and functional connectivity metrics induced by rTMS. For this purpose we primarily focused on an inhibitory 1-Hz rTMS protocol applied over the primary motor cortex. The study was conducted on young, healthy males with the intent to minimize compounding the data with uncertain hormonal effects, as well as to avoid unknown risks to the unborn fetus of prolonged rTMS and multiple 7 T MRI sessions combined in one study visit.

1-Hz rTMS over the primary motor cortex had variable influence on RMT. While 4 participants showed the expected increase in threshold, 3 participants showed a decrease in threshold. This variability is expected given the known variability in response amplitude and polarity to rTMS across individuals (Lopez-Alonso et al., 2014; Huang et al., 2017).

On the other hand, the quality of acquired ^1^H MR spectra resulted in neurochemical profiles of high inter-subject and intra-session reproducibility. High inter-session reproducibility of spectra acquired at 7 T with semi-LASER was demonstrated in previous studies by our group (Bednařík et al., 2018; Cheong et al., 2019). In order to minimize biases introduced by VOI compositions or by relaxation effects, the MRS post-processing pipelines included the different water content of GM, WM, and CSF fractions in each dataset (Gasparovic et al., 2006). For the first time, the pipelines also included the signal amplitude correction applied on metabolite basis spectra to account for differences in T_2_-relaxation between CH_3_ groups and other protons in the same molecule. Ultimately, the entire acquisition and processing strategy allowed the reliable quantification of an extensive set of neurochemicals. Of particular note is the quantification of GABA. The average CRLB (13%) and across-subjects coefficient of variation (15%) obtained with semi-LASER were similar to those observed with edited-MRS sequences at 7 T (Chen et al., 2017; Hendriks et al., 2018). A direct comparison of acquisition strategies in same subjects is still warranted to ultimately identify the most accurate and sensitive approach for GABA detection in functional MRS designs. Yet, the results of the present study indicate that semi-LASER can be suitable to detect small functional changes in GABA levels, despite the large overlaps of other resonances typical of non-edited MRS approaches. In fact, whereas handling of macromolecules can have major effects on metabolite quantification, especially GABA, the inclusion of MM spectrum in the LCModel basis set resulted in high precision of MM content assessments, and minimized the negative correlation between GABA and MM quantification. The absence of strong negative correlations between GABA and any other metabolite quantified in LCModel further supports a reliable estimate of GABA with short echo-time semi-LASER sequence. Trends of small rTMS-induced changes were ultimately detected in GABA levels of both ipsilateral and contralateral motor cortices. The increase in GABA concentration post-rTMS in the ipsilateral cortex, along with its decrease in the contralateral cortex, were in line with our initial hypothesis on GABA. However, the creatine and aspartate findings in the contralateral cortex were unexpected, and the explanation has not been resolved yet. In addition, the hypothesized decrease in glutamate levels in the contralateral motor cortex post-rTMS was not detected in this study despite the exceptional accuracy and reliability of glutamate quantification at 7 T as confirmed by an average CRLB of only 2%. Moreover, some unexpected differences in metabolite concentrations, including GABA and glutamate among others, were observed at baseline (i.e., pre-rTMS) between left and right motor cortices. The lack of randomization in the order of left and right MRS acquisitions unlikely explains such finding, as there are no conceivable dynamic events that may compound concentrations of selected metabolites in the absence of tasks or interventions. If corroborated by future research, the inter-hemispheric difference of neurochemical profiles would be interesting and informative, as it may uncover neurochemical imbalances that are potentially instrumental to support dominant and non-dominant hemisphere functions. However, further studies are necessary to specifically evaluate if and to what degree was the data acquisition affected by the chemical shift displacement error in the same direction. Therefore, acquiring MRS data with opposite gradient polarity would be helpful to eliminate possible biases.

Our finding of increased GABA concentration after 20 min of inhibitory 1-Hz rTMS in the dominant motor cortex are similar to what reported by Marjanska et al. (2013), who also found a mild GABA increase after 8 min of excitatory 5-Hz rTMS in healthy volunteers by using edited MRS at 3 T for selective detection of NAA, Glx and GABA. The finding also resembled the GABA increase measured 20 minutes after 40-s of continuous theta burst stimulation (Stagg et al., 2009b). While there is a relative paucity of studies employing MRS and rTMS interventions applied to the motor cortex, metabolic changes in such brain areas have been more extensively studied in correspondence of transcranial direct current stimulation (tDCS), which is another form of neuromodulation that utilizes constant, low direct current delivered via electrodes to the head. Similar to high vs. low frequency rTMS protocols, the anodal and cathodal forms of tDCS increase or decrease neuronal excitability, respectively. In particular, anodal tDCS applied over the motor cortex using various protocols has been found to cause significant decrease in GABA concentration in the ipsilateral motor cortex (Stagg et al., 2009a; Kim et al., 2014; Bachtiar et al., 2015, 2018; Antonenko et al., 2019; Patel et al., 2019). Few tDCS MRS studies have been conducted at 7 T on the same premise of the present study that sensitivity and reliability of metabolic measurements largely benefit from ultra-high magnetic fields (Stagg et al., 2009a; Kim et al., 2014; Ryan et al., 2018). Yet the results of these tDCS 7 T investigations have been variable. No changes in Glu or GABA were observed in correspondence of bihemispheric tDCS (Ryan et al., 2018), while cathodal stimulation was reported to either induce a significant decrease in Glu/Cr (Stagg et al., 2009a) or no observable Glu change (Kim et al., 2014).

Functional changes in glutamate levels *during* sensory stimulation are generally ascribed to increased energetic demands, rather than to variations of the (small) synaptic pool due to increased neurotransmission (Mangia et al., 2009). However, the link to energetics likely does not apply for study designs investigating metabolite changes 20–30 min *after* a prolonged neuromodulatory interevention, at which point the energy balance has concivably gone back to baseline. In this case, it is rather plausible that changes in glutamate and/or GABA levels result from the sustained neuromodulatory effects of the intervention *per se*. Indeed, rTMS produce changes in ipsilateral corticomotor excitability lasting 15–60 min post-stimulation in healthy adults (Chen et al., 1997; Hoogendam et al., 2010). Thus, inhibitory (1 Hz) and excitatory (5 Hz) rTMS protocols would be expected to substantially increase the pools of GABA and glutamate concentrations, respectively. This timeframe may allow for a more reliable signal and the detection of changes within the synaptic pool. The fact that changes in neurotransmitter levels observed after neuromodulatory interventions are not related to energetics is consistent with the lack of concomitant changes in lactate and glucose that are typically observed during external stimuli.

Our data do not support changes in T2^∗^ 30–60 min after a 20-min long rTMS intervention, as the spectra line-widths of the post-assessments were not different from those of the pre-assessments. On the other hand, fMRI signal changes due to T2^∗^ alterations are commonly observed during or shortly after TMS of the motor cortex (Liew et al., 2014 and reference therein; Navarro de Lara et al., 2017), consistent with the acute effects of TMS on cortical excitability. The marked differences in acquisition protocols and assessment timings, along with the limitations of prolonged MRS acquisitions to detect T2^∗^ changes, may explain the differences in results. In fact, it should be emphasized that our study was not designed to quantify T2^∗^, and spectra LW may be compounded by imperfect voxel re-positioning and changes in shimming during the acquisition.

No rTMS-induced changes were observed in any of the metrics measured to characterize local and distant connectivity of the motor cortices, possibly due to the prolonged delay of the post-assessment from the rTMS intervention. In a previous study by Watanabe et al. (Watanabe et al., 2014) connectivity between bilateral primary motor cortices was found to be higher 10 minutes after 30-min of inhibitory 1-Hz rTMS as compared to the inter-hemispheric connectivity measured 1 week before the rTMS session, however, longer aftereffects were not investigated. Another study by Ji et al. (2017) found that, whereas 30 min of inhibitory 1-Hz rTMS increased connectivity in the left paracentral gyrus, the effect lasted only up to 10 min after the intervention. Despite the lack of statistically significant connectivity changes after rTMS, negative correlations were found between percent changes of few connectivity metrics in both hemispheres and GABA changes in contralateral motor cortex. The implications of these relationships should be validated and explored with future studies in larger cohorts. Interestingly, a negative correlation between GABA and functional connectivity was also previously noticed in motor cortex during tDCS (Stagg et al., 2014; Bachtiar et al., 2015).

### Limitations

This pilot study was limited by the small number of participants, therefore no firm conclusions can be drawn until the findings are confirmed in larger cohorts. Yet our preliminary GABA findings are promising and consistent with common understanding of the mechanisms of rTMS as well with some previous literature. Calculations of sample sizes for future studies can be based on the findings of the present pilot investigation by carefully accounting for multiple comparison corrections according to the number of intended primary outcomes and general study design (e.g., number of regions of interest). Another limitation was the timing between the neuroimaging and neuromodulatory intervention. To avoid unforeseen problems related to the use of rTMS inside the 7 T magnetic field, we administered rTMS outside the scanner. The careful evaluation of the neurophysiology of the subject after rTMS, along with the optimization procedures needed for MRS acquisitions, led to 32, 59, and 84 min delays on average of the post-assessments MRS in left and right motor cortices, and rsfMRI, respectively. Despite this substantial delays, we could still detect rTMS-induced changes in GABA, although the delay was most likely too long for observing changes in functional connectivity. Simultaneous TMS/fMRI acquisitions at 3 T have been recently reported in the literature by implementing a dedicated multi-channel coil (Navarro de Lara et al., 2017), and such approach may be used for monitoring more efficiently the acute effects of rTMS on functional connectivity. However, the safety of TMS inside a 7 T scanner has not yet been shown, and would thus need to be established with future studies. The single-voxel MRS performance of a dedicated coil for simultaneous TMS/MRI would also need to be evaluated separately.

### Conclusion

We conclude that single-voxel 7 T MRS with semi-LASER allows safe and reliable measurements of rTMS-induced changes in the full neurochemical profiles of motor cortices. GABA changes were detected with statistical significance after the rTMS despite the small sample size of 7 subjects and despite prolonged delays from the rTMS. On the other hand, post-assessment timing needs to be further optimized to allow detection of putative functional connectivity changes. Future investigations exploring the correlation between neuromodulation and neuroimaging are indicated, in concert with the influence on motor function and outcomes. Such investigations open tremendous opportunities for understanding the mechanism of actions of neuromodulation strategies by characterizing the mediation of key neurotransmitters such as GABA and glutamate. They also hold promise in discovering potential biomarkers for responders to neuromodulatory interventions, and optimizing functional outcomes.

## Data Availability Statement

The datasets generated for this study are available on request to the corresponding author.

## Ethics Statement

The studies involving human participants were reviewed and approved by the Institutional Review Board: Human Subjects Committee of the University of Minnesota. The participants provided their written informed consent to participate in this study.

## Author Contributions

HG and BG participated in design of the work, acquisition, analysis and interpretation of the data, and preparing the manuscript. IT participated in analyses and interpretation of the data, and preparing the manuscript. PB participated in acquisition and analyses of the data, and editing the manuscript. DM and DD participated in analyses of data, and preparing the manuscript. SMi participated in design of the work, interpretation of the data, and editing the manuscript. GM and ML-M participated in acquisition of the data, interpretation of the data, and editing the manuscript. CM participated in design of the work, acquisition and analysis of the data, interpretation of the data, and editing the manuscript. LE participated in analysis of the data, interpretation of the data, and editing the manuscript. SMa participated in design of the work, analysis and interpretation of the data, and preparing the manuscript.

## Conflict of Interest

The authors declare that the research was conducted in the absence of any commercial or financial relationships that could be construed as a potential conflict of interest.

## Footnotes

1 https://www.fil.ion.ucl.ac.uk/spm/
